# Application of multiparametric MR imaging to predict the diversification of renal function in miR29a-mediated diabetic nephropathy

**DOI:** 10.1038/s41598-021-81519-7

**Published:** 2021-01-21

**Authors:** Chia-Hao Su, Yung-Chien Hsu, Suresh Thangudu, Wei-Yu Chen, Yu-Ting Huang, Chun-Chieh Yu, Ya-Hsueh Shih, Ching-Jen Wang, Chun-Liang Lin

**Affiliations:** 1 Institute for Translational Research in Biomedicine, Kaohsiung Chang Gung Memorial Hospital, Kaohsiung, Taiwan; 2 Department of Biomedical Imaging and Radiological Sciences, National Yang Ming University, Taipei, Taiwan; 3 Department of Nephrology, Chang Gung Memorial Hospital, 6 West, Chia-Pu Road, Putzu City, Chiayi Taiwan; 4 Kidney Research Center, Chang Gung Memorial Hospital, Taipei, Taiwan; 5 College of Medicine, Chang Gung University, Taipei, Taiwan; 6 Department of Medical Research, Center for Shockwave Medicine and Tissue Engineering, Kaohsiung, Taiwan; 7 Department of Orthopedic Surgery, Chang Gung Memorial Hospital, Kaohsiung, Taiwan

**Keywords:** Imaging, Nephrology, Chronic kidney disease

## Abstract

Diabetic nephropathy (DN) is one of the major leading cause of kidney failure. To identify the progression of chronic kidney disease (CKD), renal function/fibrosis is playing a crucial role. Unfortunately, lack of sensitivities/specificities of available clinical biomarkers are key major issues for practical healthcare applications to identify the renal functions/fibrosis in the early stage of DN. Thus, there is an emerging approach such as therapeutic or diagnostic are highly desired to conquer the CKD at earlier stages. Herein, we applied and examined the application of dynamic contrast enhanced magnetic resonance imaging (DCE-MRI) and diffusion weighted imaging (DWI) to identify the progression of fibrosis between wild type (WT) and miR29a transgenic (Tg) mice during streptozotocin (STZ)-induced diabetes. Further, we also validate the potential renoprotective role of miR29a to maintain the renal perfusion, volume, and function. In addition, Ktrans values of DCE-MRI and apparent diffusion coefficient (ADC) of DWI could significantly reflect the level of fibrosis between WT and Tg mice at identical conditions. As a result, we strongly believed that the present non-invasive MR imaging platforms have potential to serveas an important tool in research and clinical imaging for renal fibrosis in diabetes, and that microenvironmental changes could be identified by MR imaging acquisition prior to histological biopsy and diabetic podocyte dysfunction.

## Introduction

Diabetic nephropathy (DN), also known as chronic kidney disease (CKD), is remains one of the most leading causes of reduced lifespan in diabetic patients^1^. Even in the early stage of CKD, confer a substantial increase in the risk of cardiovascular disease (CVD)^2^. Moreover, estimated diabetic patients by 2030 is expected to be ~ 450 million and economical cost is projected around $490 billion/year^3^. Importantly, the main pathological features of DN are renal function and its structural changes including renal interstitial fibrosis, albuminuria, glomerular basement membrane thickening, glomerular hypertrophy, tubular hypertrophy, and podocyte injury^4^. Specifically, the main indictor to predict the worsening of kidney function is degree of renal fibrosis, due to the accumulation of proteins such as collagen and fibronectin. Another important factor of DN is gradual damage of glomerular podocytes which significantly results the leaking of proteins into the urine^5^. Clinically, blood and urine analysis have been employed to diagnosis the kidney disorders but these results are insufficiently sensitive for an effective diagnosis. Subsequently, several biomarkers were identified based on the glomerular and tubular damage^6^. Some examples such as measurement of albumin excreted in the urine, Excretion of urinary non-albumin proteins, increased urinary transferrin, estimation of Kidney injury molecule 1 (KIM-1), increased Fibronectin (FN), increased levels of Retinol-binding protein (RBP), Redox-regulating protein p66Shc, Apolipoproteins and some Serum/plasma biomarkers, etc. However, by considering the sensitivity/accuracy of results still there is an emerging/novel approach such as therapeutic or diagnostic are highly desired to conquer the CKD at earlier stages.

Recently, medical imaging as a diagnostic tool is paying much more attention to identify the various disease models. However, choosing a right imaging technique for a specific condition is a critical issue. Molecular imaging approaches such as positron emission tomography (PET)^7^ and single photon emission computed tomography (SPECT)^8^ are both rooted in nuclear medicine. Others include magnetic resonance imaging (MRI)^9^ based on the interaction of RF signals and tissue, ultrasound^10^ which processes acoustic waves reflected off tissue, and optical systems^11^. More importantly, MRI is one of the most effective tools in medicine, allows clinicians to non-invasively obtain anatomic and metabolic/functional information with high spatial and temporal resolution^12^. In addition, it provides detailed tissue structure, physiology, metabolism and activities during disease progression. Moreover, dynamic contrast enhanced (DCE)-MRI depicts the physiological alteration and morphologic changes, and can be used to describe blood flow and tissue permeability^13^. As well, DCE-MRI signal change reflects blood vessel perfusion in tissue, and can indicate the functional and dynamic transition of contrast medium through the kidney during renal fibrosis^14^. Gadolinium (Gd) based contrast medium can describe the function of perfusion and glomerular filtration in renal tissue by DCE-MRI, but these Gd-based contrast mediums risk the development of nephrogenic systemic fibrosis in patients with renal disease^15^. Thus, to avoid using Gd-based contrast agent for renal failure and fibrosis disease, diffusion weighted imaging (DWI) tracks the random motion of water molecules in the body without the use of a contrast medium. However, the impact of intravascular water diffusion on the DWI signal is dependent on the type of tissue, and the extent to which water diffusion is restricted in biologic tissue is negatively correlated to tissue cellularity and cell membrane integrity^16–18^. Water molecule motion is further limited in tissues with a high degree of cell membrane intactness, which increases cellular density (e.g., brain edema, tumor tissue). Lipophilic cell membranes limit water molecule movement in both extracellular and intracellular space. An environment with lower cellular density offers increased extracellular space for water molecule diffusion, allowing these molecules to cross defective cell membranes from extracellular to intracellular space. Referring to DWI, the signal’s directional variation and the mean diffusivity of water molecules is called the apparent diffusion coefficient (ADC)^19^ which is the diffusion coefficient derived from the orthogonal DWI and from the average of three eigenvalues from the diffusion tensor and the mean diffusivity within the MR voxel. The ADC could be quantified to reflect the cellularity. Increased extracellular space allows for increased water mobility, as in cell necrosis and apoptosis. Previous studies have shown a correlation of water movement and mobility with water contents, cell density, and tissue edematous^20^. In addition, the ADC value could indicate the degree of renal function impairment in chronic kidney disease^21,22^. Furthermore, previous studies have noted the relationship between decreased ADC and renal fibrosis^23,24^.

Here we successfully developed a Multiparametric MRI tool to Predict the Diversification of Renal Function in miR29a-mediated Diabetic Nephropathy. We systemically employed the DCE and DWI-MR imaging techniques to study the in vivo renal properties of wild-type and miR29a TG mice during diabetes induction. Besides, we also performed the immunohistomorphometry assays to identify the expression of Masson’s trichrome staining, TGF-β1, VEGF and CD31 and compare with the control groups. Overall, the present results suggest that combining the immunohistomorphometry and multiparametric MR imaging results strongly suggests that MR molecular imaging could reflect the variation of microenvironments and vascular permeability at early stage renal fibrosis during STZ-induced diabetes.

## Results

### Anatomic MR imaging analysis between wild-type and miR29a TG mice during diabetes induction

**Figure 1:**
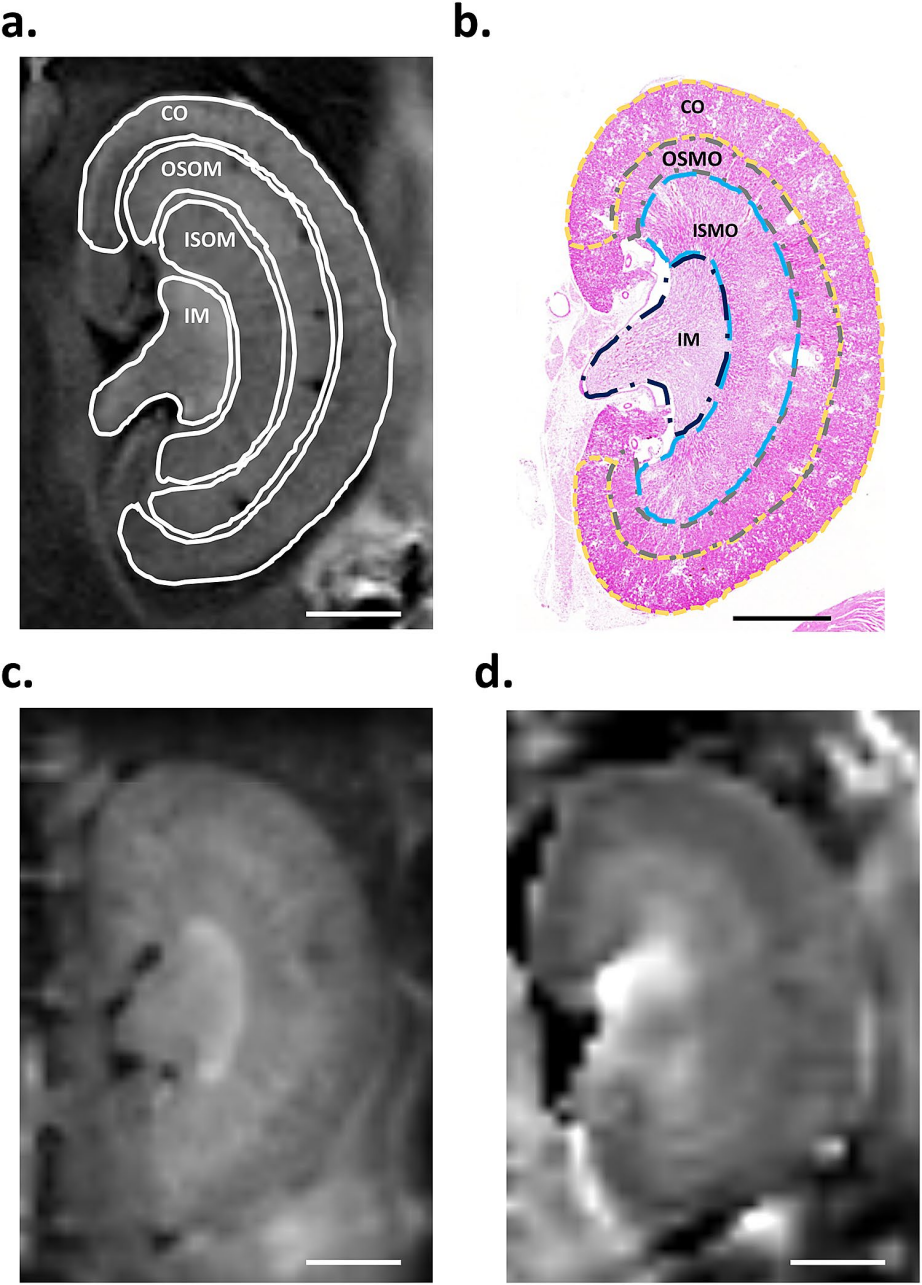
Morphological MR imaging of mouse kidney. **(a)** Coronal T2-weighted imaging, **(b)** the H&E staining of kidney, **(c)** b0 imaging, and **(d)** ADC mapping imaging form diffusion weighted imaging in a WT mouse. Renal architecture would be classified in renal cortex (CO), outer stripe of the outer medulla (OSOM), inner stripe of the outer medulla (ISOM), and inner medulla (IM) with H&E staining and T2-weight imaging. (The scale bar = 2000 µm).

**Figure 2:**
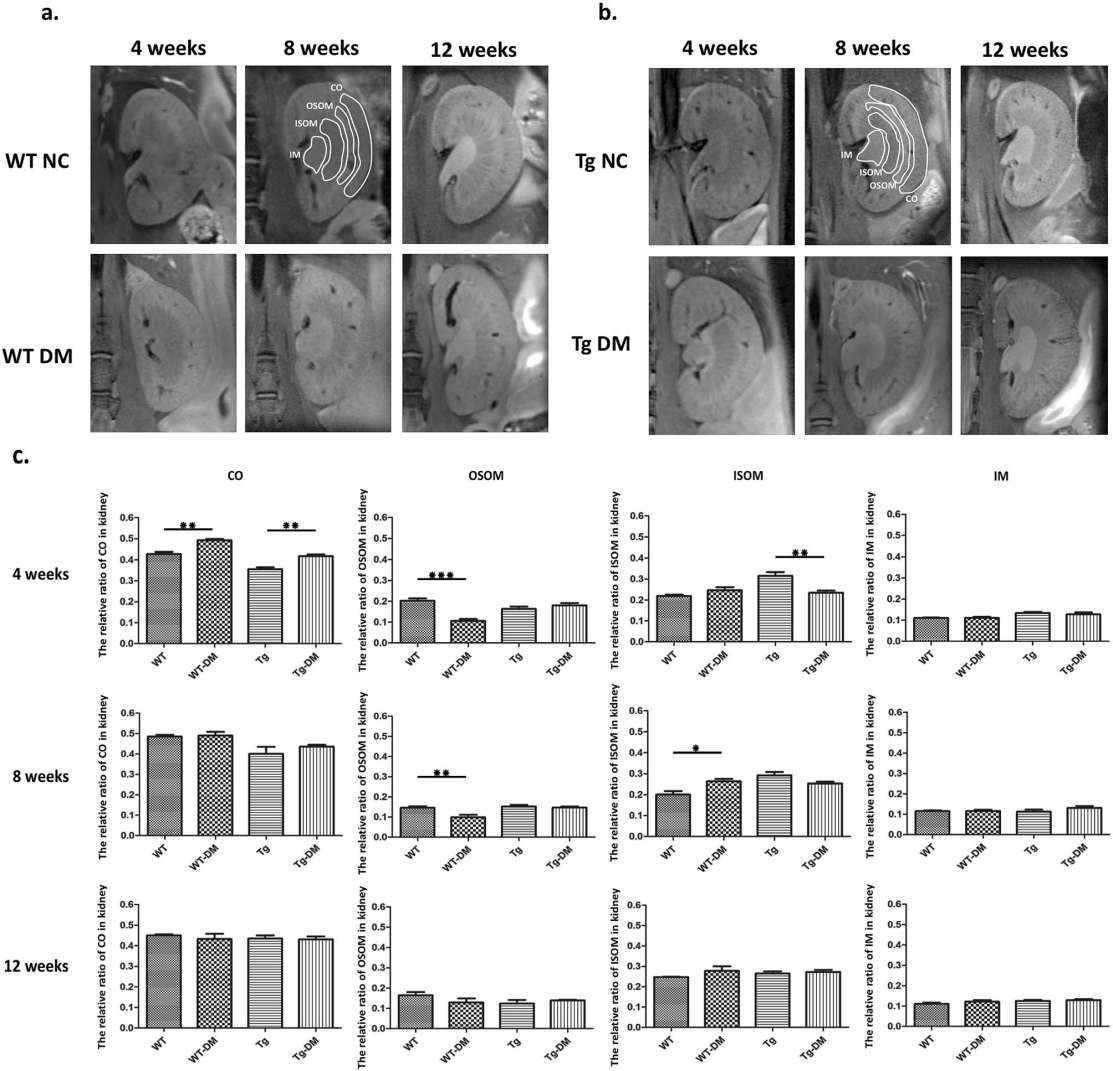
Kidney morphologies in wild-type and miR29a transgenic mice with the control and DM animals. Coronal T2-weighted imaging of renal architecture in **(a)** WT NC and WT DM; **(b)** Tg NC and Tg DM. **(c)** Ratio of renal structures in control and STZ induced DM with time variation. Data are analyzed and expressed as the mean ± SEM calculated from six mice at each time point. ** < 0.01 and *** < 0.001 with one-way ANOVA analysis, significant difference between each group. WT, wild-type; Tg, miR29a transgenic mice; NC, normal control; DM, diabetic mice. ROIs of Renal architecture with renal cortex (CO), outer stripe of the outer medulla (OSOM), inner stripe of the outer medulla (ISOM), and inner medulla (IM).

MR imaging has the benefit of a high spatial resolution and high contrast differentiation between soft tissues, which enables the simultaneous extraction of physiological, molecular and anatomical information. Herein, we used a high magnetic field MR imaging system to observe the fine structure between wild-type (WT) and miR29a Tg (Tg) mice during the induction of diabetes. To make it more clear, firstly we calculated the ratio of the renal architecture (i.e., the ratio of kidney layer % = voxel of layer/total voxel of kidney) by delineated the region of interest (ROI) with manual contouring (Fig. 1a) on advice of nephrologist, also co-assistance from the immunohistochemistry (IHC) staining analysis (shown in Fig. 1b).From the high-resolution coronal T2-weighted MR imaging (T2-WI) we could identify the structure of the renal cortex (CO), the outer stripe of the outer medulla (OSOM), the inner stripe of the outer medulla (ISOM), and the inner medulla (Fig. 1c) without motion artifacts. Furthermore, we also demonstrate that the diffusion weighted imaging (DWI) with ADC mapping imaging (Fig. 1d) are distortion-free and the layers of kidney were clearly distinguishable, as compared with T2-WI. As shown in Fig. 2a,b, we can identify the individual layers of the renal structures, and anatomic imaging did not exhibit obvious change. We analyzed the ratio of the renal architecture in control and STZ-induced diabetes groups between WT and Tg mice at 4, 8, and 12 weeks (shown in Fig. 2c). The ratio of CO increased significantly in the STZ-induced diabetes groups at 4 weeks, including increases from 0.43 ± 0.03 (WT) to 0.49 ± 0.01 (WT-DM) and 0.036 ± 0.02 (TG) to 0.42 ± 0.02 (TG-DM), respectively, for the WT and miR29a TG groups. In addition, the CO showed no variation at 8 weeks or 12 weeks between the control and STZ-induced diabetes groups. On the other hand, in the OSOM layer, the ratios of WT-DM were markedly lower than for the control group in different weeks following STZ. The ratio decreased from 0.20 ± 0.11 to 0.11 ± 0.02, followed by a further decrease at 8 weeks (from 0.15 ± 0.02 to 0.10 ± 0.02). Contrarily, the miR29a Tg mice showed no such change in the OSOM layer. Furthermore, the ratio of ISOM shows that the area of the WT-DM groups increased significantly at 8 weeks (from 0.20 ± 0.04 to 0.26 ± 0.02). Notably, the ratios of ISOM in the Tg groups differed from the WT groups, where the Tg-DM group decreased compared against the Tg control at 4 weeks. The IM renal structure showed no change during the STZ-induction of diabetes in each group, and all renal structures (i.e., CO, OSOM, ISOM, and IM) showed no significant variation at 12 weeks, despite changes to kidney weight and urinary proteins.

### Renal functional dynamic contrast enhancement MRI (DCE-MRI) for in vivo renal properties

DCE-MRI is based on T1-weighted imaging, and can assess dynamic signals caused by gadolinium-based contrast agents that would transit through the renal cortex, medulla, and collecting system. In addition, DCE-MRI also has potential to reveal renal properties. Figure S1 shows the anatomic coronal section (Figure S1a) and the nephron circulation between the cortex and medulla (Figure S1b). Subsequently, we applied the DCE-MRI to evaluate the renal filtration during STZ-induced DM of WT and Tg mice at different times. MR signals can be affected by many MR signal artifacts, such as inflow effect, signal dephasing, partial-volume effect, and flow pulsation. First, we demonstrate the impact of the inflow artifact in our system by calculating the arterial input function (AIF). In Figure S2, we delineate the ROI with manual contouring at different levels of the aorta (Figure S2a), finding that the AIFs are similar at different aorta regions (Figure S2b). This indicates that the DCE-MRI signals are not affected by inflow in our system. To evaluate the renal function of perfusion during STZ-induced DM, we calculate the K^trans^ using DCE-MRI. K^trans^ is a measure of capillary permeability, and was calculated by measuring the accumulation of gadolinium-based contrast agent in the extravascular-extracellular space.

**Figure 3:**
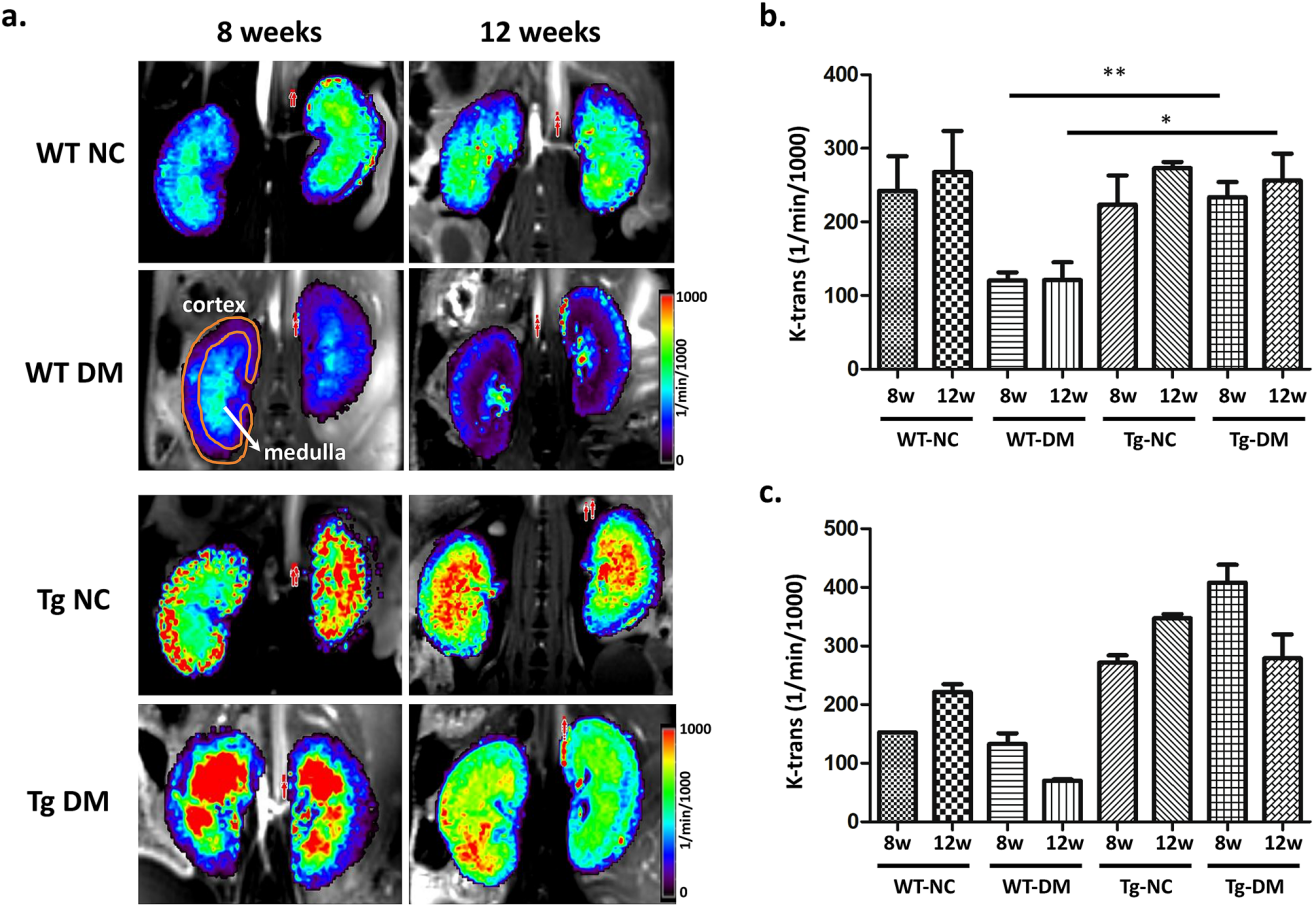
Comparison of DCE-MRI for wild-type and miR29a transgenic mice as the control and DM animals. **(a)** DCE-MR imaging with K^trans^ mapping of kidneys in each experimental group from vascular-phase images, and the unit of the color bar is mL/min/1000 mL; **(b)** value of K^trans^ in renal cortex structures with control and STZ induced DM groups; **(c)** value of K^trans^ in renal medulla structures with control and STZ induced DM groups. (* < 0.05 with one-way ANOVA analysis, significant difference between each group; WT, wild-type; Tg, miR29a transgenic mice; NC, normal control; DM, diabetic mice; n = 5).

Based on the DCE-MRI, we analyzed the K^trans^ values of the cortex and medulla during STZ-induced DM of WT and Tg mice. K^trans^ showed no significant change among the various groups at 4 weeks. Figure 3 shows the K^trans^ values at 8 and 12 weeks, wherein the cortex exhibits a higher flow of WT-NC than WT-DM (upper part of Fig. 3a). Contrarily, the regions of extremely high flow reflect the renal perfusion in the cortex of miR29a Tg mice during STZ-induced DM.

Besides, we also calculated the K^trans^ value in the renal cortex and medulla (shown in Fig. 3b and c). Results show that inducing diabetes in WT mice decreases the K^trans^ value in the renal cortex from 242.3 to 120.5 and 267.8 to 121.1 mL/min/1000 mL, respectively, at 8 and 12 weeks. On the other hand, no significant change was found in the cortex of Tg-CN and Tg-DM mice. In addition, the medulla also exhibited similar variation between WT-NC and WT-DM mice, and K^trans^ decreased drastically at 12 weeks (from 227.8 to 70.4 mL/min/1000 mL). These results indicate that the kidney perfusion was affected in WT mice and that capillary permeability worsened 8 and 12 weeks following STZ-induced diabetes.

In our previous work, we reported the restoration of miR-29a signaling by miR-29a transgenic mice alleviated diabetic podocyte dysfunction and mesangial fibrosis^25,26^. Therefore, miR-29a transgenic mice were used to investigate and confirm whether gain of miR-29a signaling could rescue renal fibrosis, perfusion and angiogenesis. Compared to wild-type mice, we found that miR-29a transgenic mice were resistant to developing significant albuminuria following STZ treatment^25,26^. Masson’s trichrome and TGF-β staining showed more severe glomerular and tubule-interstitial fibrosis in the kidneys of diabetic mice. We further found that miR-29a transgenic mice treated with STZ displayed lower levels of fibrosis when compared to those of wild type diabetic kidneys (Figure S3 and S4). Figure S5 shows the signals of Masson’s trichrome and TGF-β staining. Comparison between groups shows the glomerulus and tubule of WT-DM increased significantly in TGF-β and Masson’s trichrome staining, indicating that the fibrosis level increased during STZ-induced diabetes, and K^trans^ in the renal cortex and medulla was negatively correlated with fibrosis (shown in Figures S5a and S5b). On the other hand, we also found that the fibrosis of the glomerulus and tubule increased slightly in Tg-DM (shown in Figures S5c and S5d), but miR-29a transgenic mice were resistant to developing significant albuminuria following STZ treatment, and vascular permeability could be maintained as normal.

### Evaluation of the mobility of tissue water molecules in STZ-induced diabetes with diffusion MRI

DWI is used to track the random motion of water molecules in the body. Outside the body, water molecules move in constant random Brownian motion, a phenomenon referred to as free diffusion. However, in biologic tissue, the movement of water molecules is restricted by interactions with cell membranes and macromolecules.

The directional variation in signal and the mean diffusivity of water molecules is termed ADC. Previous studies have shown correlations among water contents, cell density in tumor mass, and tissue edematous. ADC is the diffusion coefficient obtained from orthogonal diffusion weighted MRI and derived from the average of three eigenvalues from the diffusion tensor and the mean diffusivity within the MR voxel. In addition, the ADC value could indicate the degree of renal function impairment due to chronic kidney disease. Previous studies have noted the relationship between decreased ADC value and renal fibrosis. In diabetic animal models, edematous cellular damage would affect the variation of ADC values. In the analysis of renal architecture, the ratio of tissue volume exhibited significant differences in CO, ISOM, and OSOM between 4 and 8 weeks, respectively. In addition, the renal fibrosis resulted in no difference to the ratio of renal structures at 12 weeks, and we could not evaluate the kidney function based on the coronal T2-weighted imaging. We used DWI to evaluate the movement of water molecules in kidney microenvironment between control and DM models.

**Figure 4:**
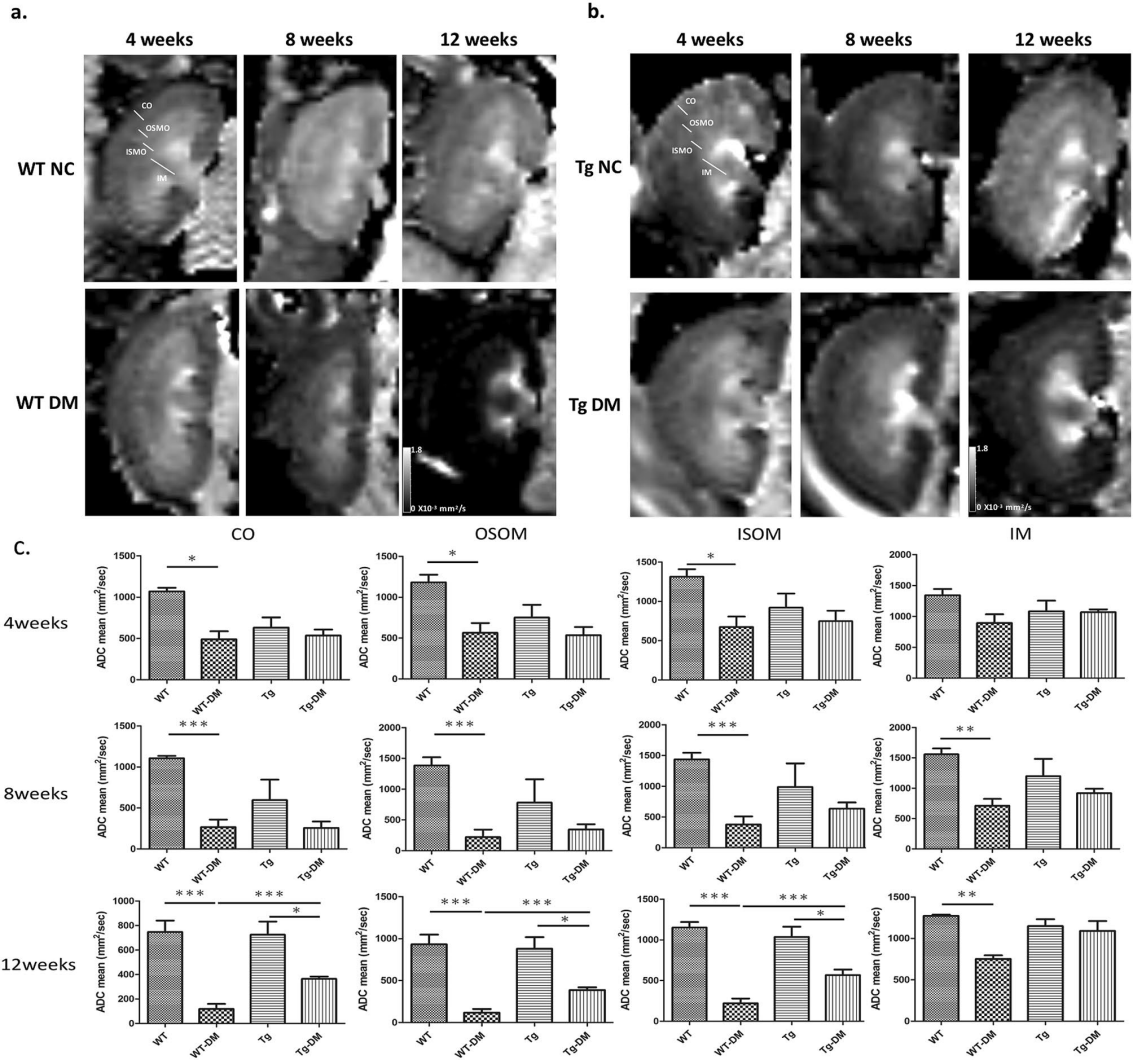
Apparent diffusion coefficient (ADC) mapping of kidneys in control and DM groups. **(a)** WT control and DM; **(b)** miR29a Tg control and DM. ADC mapping was decreased during STZ-induced DM model. ROIs were copied from b0 images to ADC mapping, and ADC values were calculated separately for all anatomic layers at different times **(c)**. (* < 0.05, ** < 0.01, and *** < 0.001 with one-way ANOVA analysis, significant difference between groups).

ADC mapping in WT and miR29a with normal renal function were higher than that in STZ-induced DM models, indicating that water molecule movement was affected by renal dysfunction (Fig. 4a,b). The relative ADC value of CO in the kidneys between WT CN and DM showed a significant decrease in the WT DM group (54.23%) that compared with the control at 4 weeks (Fig. 4c). In addition, the relative ADC value of CO respectively decreased to 75.86% and 80.83% at 8 and 12 weeks. On the other hand, miR29a Tg mice only showed a lower relative ADC value at 12 weeks, decreasing to 51.28% in CO of Tg DM group. For OSOM and ISOM, we also found the same variation with CO as in the induced diabetic mice. These results for DWI and ADC mapping indicate that miR29a Tg mice could decrease renal dysfunction in the DM model.

### miR-29a transgenic mice are resistant to renal fibrosis and proliferation of endothelial cells after induction of diabetes

Next, we investigated the effect of increased miR-29a signaling on renal angiogenesis in diabetic kidneys. High power field microscopic analysis of glomerular mesangium and tubulointerestital area further demonstrated that miR-29a transgenic mice expressed lower VEGF levels than wild-type mice after diabetes induction (Figure S6). We further used the immunohistochemical expression pattern of commonly used endothelial cells markers like CD31 to evaluate the diabetic glomerular area and tubulointerstitial area (Figure S7). Interestingly, similar to VEGF expression, we found that diabetic mice increased CD31 expression. In addition, immunohistochemical analysis revealed that gain of miR-29a in renal tissues was accompanied by the downregulation of endothelial cell factor CD 31 expression in the glomerular area and tubulointerstitial area. Following VEGF and CD31 staining, Figure S8 shows the results of signal analysis. We find that WT-DM showed the strongest signal in VEGF and CD31, indicating the proliferation of endothelial cells in the glomerulus and tubule after diabetes induction. TGF-β plays a central role in fibrogenesis by modulating the fibroblast phenotype and function. In addition, endothelial cells transdifferentiate into mesenchymal cells through a process termed endothelial-mesenchymal transition in the development of fibrotic diseases and TGF-β is upregulated with inducing endothelial-mesenchymal transition and contributes to the development of fibrosis^27^.

Thus, immunohistomorphometry results showed that diabetes significantly increased Masson’s trichrome staining, TGF-β1, VEGF and CD31 expression of glomerular and tubulointerstitial area compared with those in control group. miR-29a Tg mice significantly down-regulated Masson’s trichrome staining. TGF-β1 coincided with reduced VEGF and CD31 in the renal glomeruli and tubulointerestital area of diabetic mice.

## Discussions

Currently, quantification of fibrosis from renal injury is limited to invasive renal biopsy histology analysis. Non-invasive MR imaging has promise as an important research and clinical imaging tool for renal fibrosis in diabetes, and could be used to identify microenvironmental changes with MR imaging acquisition prior to histological biopsy and diabetic podocyte dysfunction. Firstly, based on the morphological MR imaging of the kidneys, we identified the renal structures and assessed the change of CO, OSOM, ISOM, and IM between STZ-induced diabetes in WT and TG mice. As a result, calculating the ratio of the renal architecture we could find that the volumes change in CO, OSOM, and ISOM in WT-DM at 4 or 8 weeks. Noticeably, we found volume increased in CO of the Tg-DM groups at 4 weeks. These results indicate that miR29a had a renoprotective effect during STZ-induced diabetes. As its renowned that the DCE-MRI technique is based on T1-weighted imaging, and can assess dynamic signals caused by gadolinium-based contrast agents that would transit through the renal cortex, medulla, and collecting system. In addition, DCE-MRI also has potential to reveal renal properties. Based on DCE-MRI results, we found that K^trans^ values of renal cortex and medulla were significantly decreased from 242.3 to 120.5 and 267.8 to 121.1 mL/min/1000 mL (cortex), and 227.8 to 70.4 mL/min/1000 mL (medulla) respectively, at 8 and 12 weeks in a WT-NC and WT-DM mice. These DCE-MRI results can able to distinguish the STZ-induced diabetes. Furthermore, we also observed that the fibrosis of the glomerulus and tubule increased slightly in Tg-DM whereas miR-29a transgenic mice were resistant to developing significant albuminuria following STZ treatment, and vascular permeability could be maintained as normal. Subsequently, ADC mapping of renal function in WT and miR29a were performed and the data reveals that the water molecule movement was greatly affected by renal dysfunction. Based on the correlation between ADC value of DWI and renal fibrosis, we could observe the decreasing of ADC in diffusion imaging during renal fibrosis progression. In diabetic animal models, edematous cellular damage would affect the variation of ADC values. Evaluating the expression levels of profibrotic factor TGF-β1, we consistently find that miR-29a transgenic mice treated with STZ displayed lower levels of TGF-β1 (Figures S3 and S6a, b) than wild-type STZ-treated mice. Comparison with the results of DWI and ADC mapping supports that miR29a Tg mice could decrease renal dysfunction in the DM model. Further, immunohistomorphometry assay was performed to identify the expression of Masson’s trichrome staining, TGF-β1, VEGF and CD31 and the data revels that the levels of expression were significantly higher in the diabetic mice than the control groups. Based on the results, we strongly believed that combining the immunohistomorphometry and multiparametric MR imaging results strongly suggests that MR molecular imaging could reflect the variation of microenvironments and vascular permeability at early-stage renal fibrosis during STZ-induced diabetes. Furthermore, miR-29a transgenic mice could significantly retard diabetes-induced prototype of CKD progression including the proliferation of endothelial cells and fibrosis.

## Conclusion

In summary, we successfully employed a Multiparametric MRI tool to Predict the Diversification of Renal Function in miR29a-mediated Diabetic Nephropathy. The main key findings as follows, (1) First, we found that the down-regulation of miR-29a would activate the fibrogenic markers of Masson’s trichrome and TGF-β1 in IHC staining during diabetic animals. (2) Markers of endothelial cells (VEGF and CD31 expression) were concomitantly detected in glomeruli and tubulointerestium of streptozotocin-induced diabetic mice at 8 weeks. (3) The constructed miR-29a transgenic mice that supported sustained miR-29a signaling were found to resist profibrotic and vascular permeability from streptozotocin-induced diabetes. (4) Furthermore, our findings suggest that the application of DCE-MRI and DWI could be associated with the progression of fibrosis and between WT and Tg mice during STZ-induced diabetes. (5) In addition, we validated the role of miR29a in producing a renoprotective effect to maintain renal perfusion, volume, and function. Moreover, K^trans^ of DCE-MRI and ADC of DWI were found to could reflect the level of fibrosis between WT and Tg mice. These results suggest that non-invasive MR imaging platforms have promise to support research and clinical imaging for renal fibrosis in diabetes, and the change of microenvironment could be identified with MR imaging acquisition prior to histological biopsy and diabetic podocyte dysfunction. Overall, present study also provides indications that that more intensive gene therapies and fibrosis level evaluations are needed for the management of chronic fibrosis patients.

## Experimental section

### Diabetic animal models

Briefly, intraperitoneally administered the 50 mg/kg of streptozotocin (STZ) to the Four month old male FVB mice (BioLasco Biotechnology Co., Taiwan) for inducing diabetes. According to the previously described protocol, 1–2 unit/kg insulin was given to STZ-induced diabetic mouse for equalizing the blood glucose levels^28,29^. The diabetic in animals was confirmed by its post-fasting blood glucose levels (200–300 mg/dl). Diabetic or normal animals were sacrificed with an overdose of sodium pentobarbital at 5 weeks (n = 6) after onset of diabetes. All animal experiments were approved by the Institutional Animal Care and Use Committee of Chang Gung Memorial Hospital (IACUC No. 2016070801), and were performed in accordance with the Animal Protection Law by the Council of Agriculture, Executive Yuan (R.O.C.) and the guideline of National Research Council (U.S.A.) for the care and use of laboratory animals.

### miR-29a transgenic mice

miR-29a transgenic mice (FVB/miR-29a^Tg^) were produced and maintained as described previously reported protocol^25^. Briefly, full length sequences of human PGK promoter and human miR-29a precursors were cloned into the pUSE expression vector. Thereafter, constructed miR-29a-containing DNA fragment was then successfully transferred into fertilized eggs from FVB/N mice. Subsequently, eggs were further transferred into ICR foster mothers.

### Mass trichrome and immunohistochemical stains

To evaluate glomerular damage including glomerulosclerosis and mesangial expansion with renal glomeruli, renal tissue sections were subjected to Masson's Trichrome Staining Protocol for Collagen Fibers according to manufacturer instructions (HT-15; Sigma-Aldrich). Immunohistochemical staining was conducted using antibodies against TGF-beta1 for pro-fibrogenic marker (BS1361; Bioworld Tech.), VEFG (sc-7269; Santa Cruz) and CD31 (ab9498; abcam) to evaluate the proliferation of endothelial cells in glomerulus and tubule, as well as horseradish peroxidase-3′-, 3′-diaminobenzidine kits (R&D Systems, Minneapolis, MN).

### Histomorphometry

For Histomorphometry analysis, we followed the previously described protocol^30^. Briefly, three random images of 0.75 mm^2^ from each area (3 mm^2^) were then taken under 400× magnification using a Cool CCD camera (CoolSNAP-Pro_*cf*_ Digital kit; Media Cybernetics, Silver Springs, MD, USA). Five sections of the glomerular area and tubulointestital area of each mouse were collected and further divided into five sub-areas of each section. For semi-quantifying the number of positive immune-labeled cells in the glomeruli and tubulointerestital area, Integral Optical Density (IOD) was analyzed using Image-Pro Plus 6.3 software (Media Cybernetics, Silver Spring, USA). For each mouse, we present the average of 25 sub-areas.

### The high spatial resolution T_2_-weighted micro-MR imaging

The in vivo kidney MR images were performed in normal FVB and FVB/miR-29a^Tg^ mice using the experimental diabetic model. The animals were anesthetized using isoflurane and MRI acquisition was performed using a 9.4 T MR imager (Bruker BioSpec 94/20 USR) equipped with a high-performance transmitter–receiver surface coil, with a maximal gradient strength of 600 mT/m. For T_2_-weighted imaging, the mice were anesthetized using 2% isoflurane (Abbott Laboratories, Abbott Park, IL) mixed with 100% O_2_ delivered using a veterinary anesthesia delivery system (ADS 1000; Engler). The contrast signal is obtained using a TurboRARE T_2_ pulse sequence (for axial section: TR/TE/FA, 2500 ms/28 ms/180 degree; MTX, 256 × 256 × 11; FOV, 30 × 30 × 0.5 mm^3^. For coronal section: TR/TE/FA, 2500 ms/28 ms/180∘; MTX, 256 × 256 × 11; FOV, 30 × 30 × 0.5 mm^3^), a NEX of 9, and the scanning with respiration gating. The MR imaging signal intensities were measured using ImageJ 1.50i software. The detailed MR parameters are shown in Table S1.

### In vivo diffusion-weighted MR imaging of kidney

We have established methods of the DW-MRI in mice brain stem studies by using previously reported protocols^31,32^. Herein, we modified the optimate imaging parameters for renal studies. The mice were placed and fixed by foam pads and then put into a mini quadrature coil for MRI scanning. For DWI, coronal multi-section echo-planar DW imaging was performed with the following parameters: TR = 2000 ms; TE = 20 ms; Bandwidth = 250,000; number of segment = 2; MTX = 96 × 96; FOV = 30 × 30 mm; Spatial resolution/pixel = 312 × 312 µm; slice number = 3; slice thickness = 0.7 mm; Interslice gap = 0.05 mm; Diffusion gradient duration = 2.5 ms; Diffusion gradient separation = 8 ms; B values per diffusion (s/mm^2^) = 100, 300, 500, 700, 800, 1000; NEX = 16; the scanning with respiration gating. The gradients were applied in three orthogonal directions and subsequently averaged to minimize the effects of diffusion anisotropy. Respiratory triggering was used with a minimum repetition time of 2000 ms. An echo time of 20 ms was applied to reduce motion artifacts. Section positioning was identical to that used with the axial T_2_-weighted sequence.

The ADC maps were generated by means of pixel-by-pixel linear regression analysis of the natural log of signal intensity versus *b* values. Data analysis was performed using regions of interest at different locations within the kidney. All values were reported as means ± SDs. The detailed MR parameters are shown in Table S1.

### In vivo DCE-MRI acquisition of FVB and FVB/miR-29aTg mice

We have established the methodologies of in vivo DCE-MRI acquisition with kidney analysis by using previously reported protocols^33^. DCE-MRI results were obtained 4, 8 and 12 weeks after inducing diabetes mellitus to assess the change in celluar permeability in the kidney. The mice were anaesthetized with isoflurane (3% for induction and 1.5–2% for maintenance) in O_2_ gases throughout the experiment. T2-weighted kidney reference imaging was based on the high spatial resolution T2-weighted micro-MR imaging that we have acquired. For the acquisition of DCE-MRI data, the lateral tail vein of each mouse was cannulated with a 27G butterfly catheter connected to a 1 m long line of polyethylene tubing (PE-5, 0.2 mm I.D., 0.5 mm O.D.), thereby enabling intravenous injection of the contrast agent.

DCE-MRI data were acquired using a T1 weighted, two-dimensional, fast low angle shot (FLASH) sequence with the following parameters: TR = 33 ms; TE = 1.8 ms; Bandwidth = 75,000; MTX = 112 × 112; FOV = 30 × 30 mm; Spatial resolution/pixel = 268 × 268 µm; slice number = 3; slice thickness = 0.7 mm; Interslice gap = 0 mm; NEX = 1; sampling interval of 2.7 s. Baseline images were acquired for 14 s (cross to repetitions = 5), followed by an injection of 0.1 mmol/kg of over 5 s, followed by further acquisitions, over a total time of 2 min 46 s 320 ms (repetitions = 60). The detailed MR parameters are shown in Table S1.

### Evaluation of MR imaging

The imaging processing was followed by using our previously reported protocols^33,34^. Briefly, ADC and K^trans^ values were analyzed using MIStar commercial software (Apollo Medical Imaging Technology, Melbourne, Australia). Regions of interest (ROIs) were manually located in the cortex, the outer stripe of the outer medulla (OSOM) and the inner stripe of the medulla (ISOM) of the kidney on T2-weighted images. The same ROIs were copied and pasted on the ADC mapping and DCE-MRI data for the determination of ADC and K^trans^ values, respectively. Furthermore, the individual arterial input functions (AIFs) were computed using T1 weighted-FLASH images. These AIFs were used in the Extended Tofts Model to determine K^trans^ values.

### Statistical analysis of histology and MR imaging

The statistical analyses were followed by our previously reported procedures^25,32,33,35^. All values were expressed as means ± standard errors. An independent-sample *t*-test was used to analyze the difference in the difference among normal, diabetic, and miR-29a transgenic mice. For MR imaging, statistical analysis was performed by obtaining the values at different time points by using one-way ANOVA followed by post hoc multiple comparisons with the Tukey–Kramer test. Cross-sectional studies were analyzed by using the Student *t* test at each time point. Statistical analysis was carried out by using GraphPad Prism Software (version 7.0, La Jolla California USA). A probability value < 0.05 was considered statistically significant.

## Supplementary Information

Supplementary Information.

## Abbreviations

CO: Renal cortex
OSOM: Outer stripe of the outer medulla
ISOM: Inner stripe of the outer medulla
IM: Inner medulla
MRI: Magnetic resonance imaging
DWI: Diffusion weighted imaging
ROIs: Regions-of-interest
FA: Fractional anisotropy
ADC: Apparent diffusion coefficient
DCE-MRI: Dynamic contrast enhancement magnetic resonance imaging
K^trans^: The volume transfer constant
