# Supplementary material for: Application of multiparametric MR imaging to predict the diversification of renal function in miR29a-mediated diabetic nephropathy
Source: Sci Rep. 2021 Jan 21;11:1909. doi: 10.1038/s41598-021-81519-7 (PMC7820287; doi:10.1038/s41598-021-81519-7)
Supplement: Supplementary file 1 — Supplementary Information. [file 41598_2021_81519_MOESM1_ESM.pdf]

Supplementary Information

Application of Multiparametric MR Imaging to Predict the Diversification of Renal Function in miR29a-mediated Diabetic Nephropathy

Chia-Hao Su[1,2†], Yung-Chien Hsu[3,4†] Suresh Thangudu[1], Wei-Yu Chen[1], Yu-Ting Huang[3], Chun-Chieh Yu[1], Ya-Hsueh Shih[3,4], Ching-Jen Wang[6,7], Chun-Liang Lin[3,4,5,6*]

[1]Institute for Translational Research in Biomedicine, Kaohsiung Chang Gung Memorial Hospital, Kaohsiung, Taiwan.

[2]Department of Biomedical Imaging and Radiological Sciences, National Yang Ming University, Taipei, Taiwan.

[3]Departments of Nephrology, Chang Gung Memorial Hospital, Chiayi, Taiwan.

[4]Kidney Research Center, Chang Gung Memorial Hospital, Taipei, Taiwan.

[5]Colleage of Medicine, Chang Gung University, Taiwan.

[6]Center for Shockwave Medicine and Tissue Engineering, Department of Medical Research.

[7]Department of Orthopedic Surgery, Chang Gung Memorial Hospital, Kaohsiung, Taiwan

[†] C.-H Su and Y.-C Hsu contributed equally to this work.

* Address for further correspondence:

Chun-Liang Lin, MD, PhD
Departments of Nephrology, Chang Gung Memorial Hospital
6 West, Chia-Pu Road, Pu-tzu City, Chiayi, Taiwan
Tel: 886-5-362-1000 Ext. 2339; Fax: 886-5-3623005
E-mail: linchunliang@adm.cgmh.org.tw

Table S1. The parameters of MR imaging acquisition with 9.4 T animal MR system

Parameter of imaging acquisition	T2WI-coronal	T2WI-axial	DCE-MRI	DWI
Sequence name	TurboRARE	TurboRARE	FLASH	DtiEpi
Bandwidth	62500	62500	75000	250000
RARE factor	8	8	-	-
Number of segment	-	-	-	2
Number of repetition	1	1	60	1
Repetition time (msec)	2500	2500	33	2000
Echo time (msec)	9.3	9.3	1.8	20
Effective echo time (msec)	28	28	-	-
Pulse angle	180 degree	180 degree	40 degree	-
Slices number	11	21	3	3
Slice thickness (mm)	0.5	0.5	0.7	0.7
Interslice gap (mm)	0	0	0	0.05
Field of view (mm^2)	30×30	30×30	30×30	30×30
Matrix dimension	256×256	256×256	112×112	96×96
Spatial resolution/pixel (mm^2)	117×117	117×117	268×268	312×312
Diffusion gradient duration (msec)	-	-	-	2.5
Diffusion gradient separation (msec)	-	-	-	8.0
B values per diffusion (s/mm^2)	-	-	-	100, 300, 500, 700, 800, 1000
Number of average	9	9	1	16

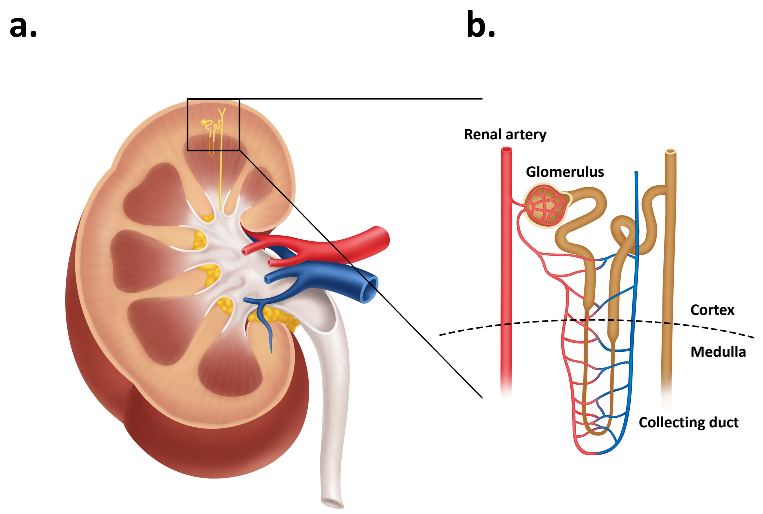

Figure S1. Illustration of mouse kidney. (a) anatomic coronal section of kidney. (b) schematic diagram of the nephron circulation between cortex and medulla.

a. **b.**

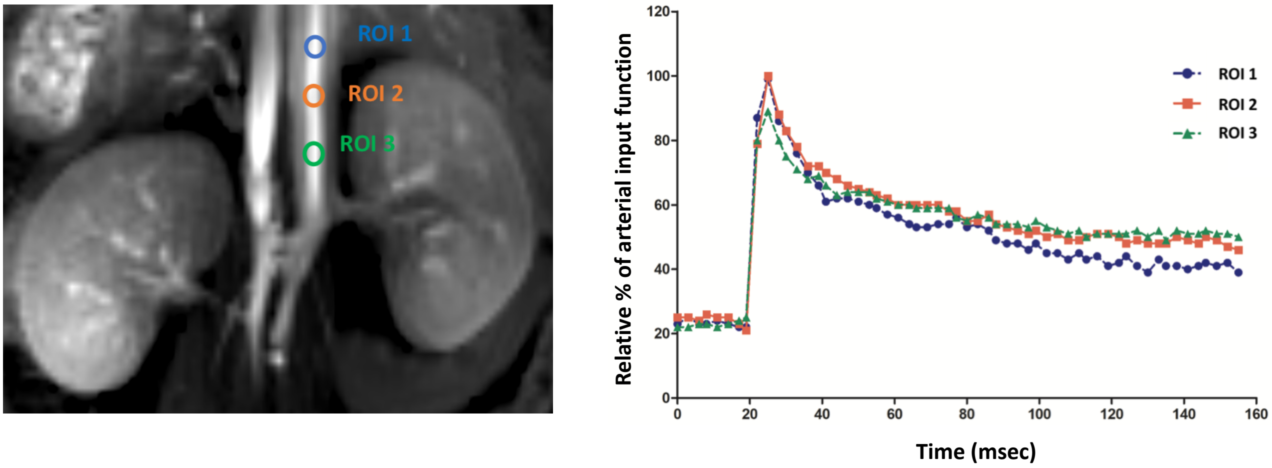

Figure S2. Variability of AIF in DCE-MRI. a) Regions of interest (ROI) at different area of the aorta, and b) the AIF of each ROI.

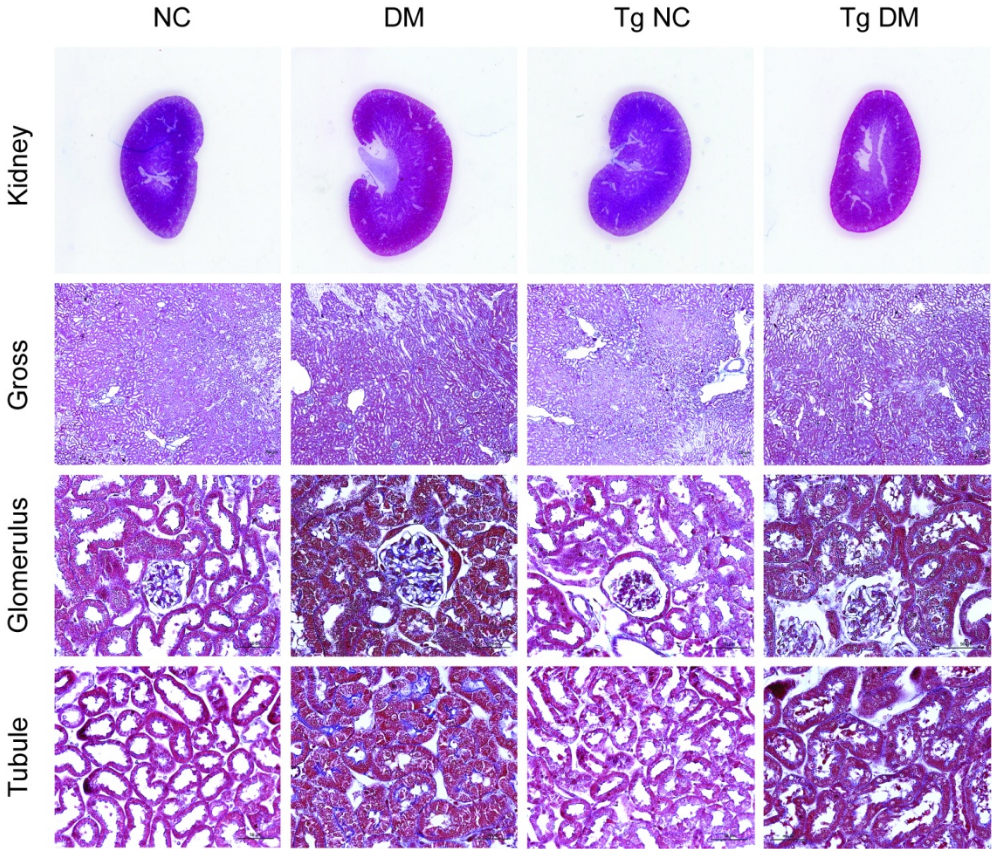

Figure S3. Representative photographs of immunohistochemical staining of fibrotic marker (Masson's trichrome) in glomeruli and tubulointerestium of wild type mice and miR-29 transgenic mice without or with STZ treatment at 8 weeks. Representative photomicrographs for Masson's trichrome staining of kidney sections in renal glomeruli and tubulointerestium of the wild-type (WT) and miR-29a transgenic mice (Tg) after induction of diabetes. NC, normal control; DM, diabetes.

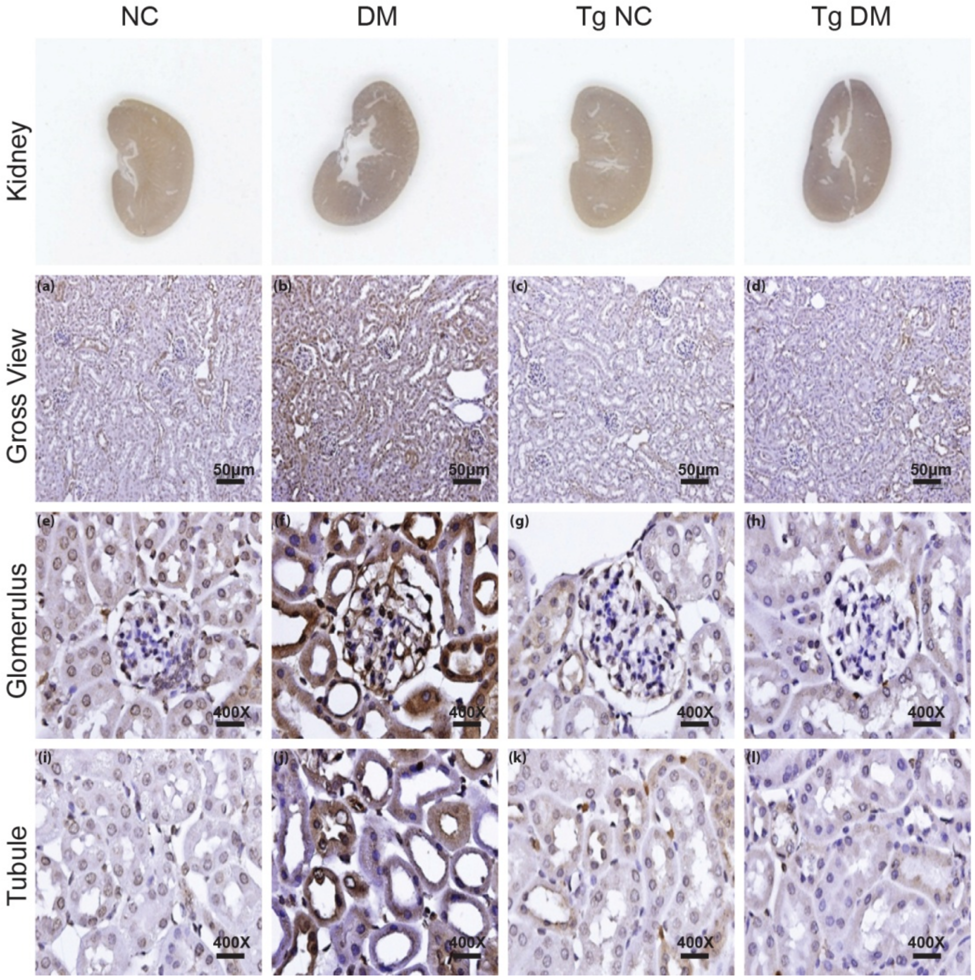

Figure S4. Representative photographs of immunohistochemical staining of pro-fibrotic marker (TGF-β1) in glomeruli and tubulointerestium of wild type mice and miR-29 transgenic mice without or with STZ treatment at 8 weeks. Representative photomicrographs for TGF-β1 staining of kidney sections in renal glomeruli and tubulointerestium of the wild-type (WT) and miR-29a transgenic mice (Tg) after induction of diabetes. NC, normal control; DM, diabetes.

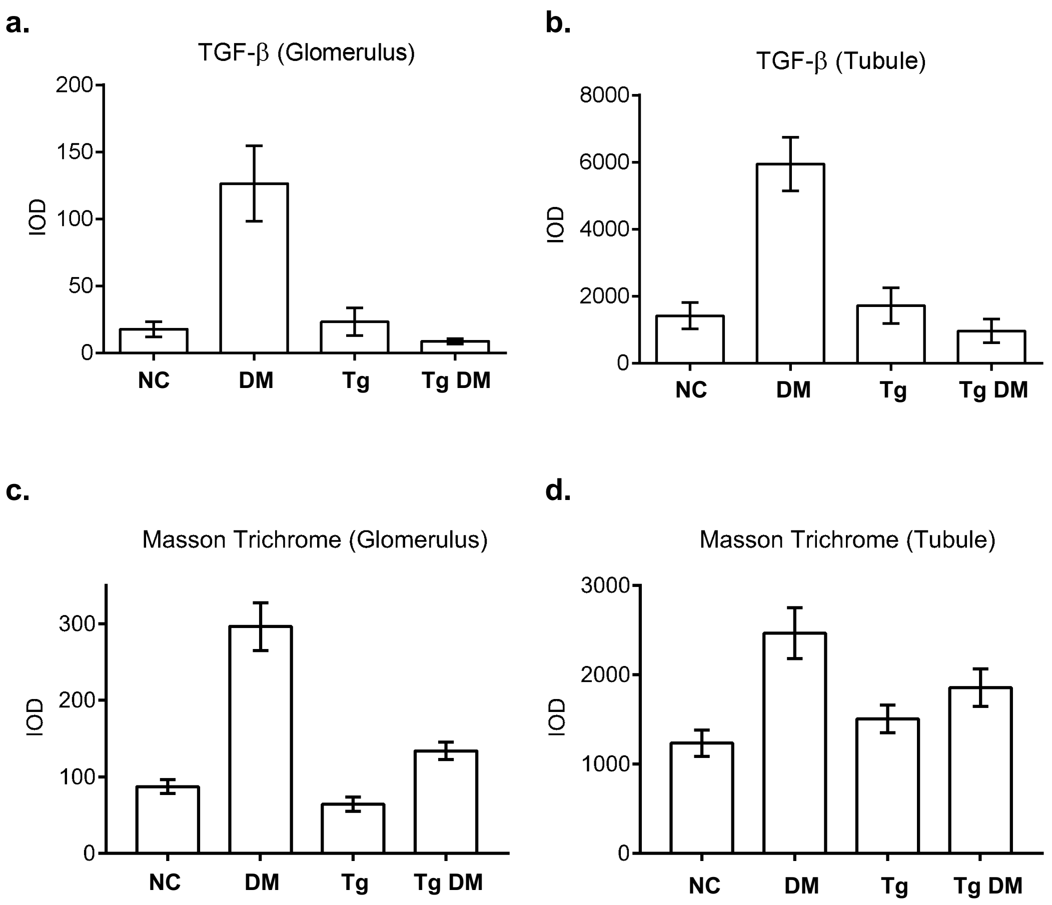

Figure S5. Quantification of fibrotic marker with TGF-β1 and Masson's trichrome in wild type mice and miR-29 transgenic mice without or with STZ treatment at 8 weeks. Quantification of TGF-β1staining of kidney sections in renal glomeruli (a) and tubulointerestium (b); quantification for Masson's trichrome staining of kidney sections in the renal glomeruli (c) and tubulointerestium (d) of the wild-type (WT) and miR-29a transgenic mice (Tg) after induction of diabetes. NC, normal control; DM, diabetes.

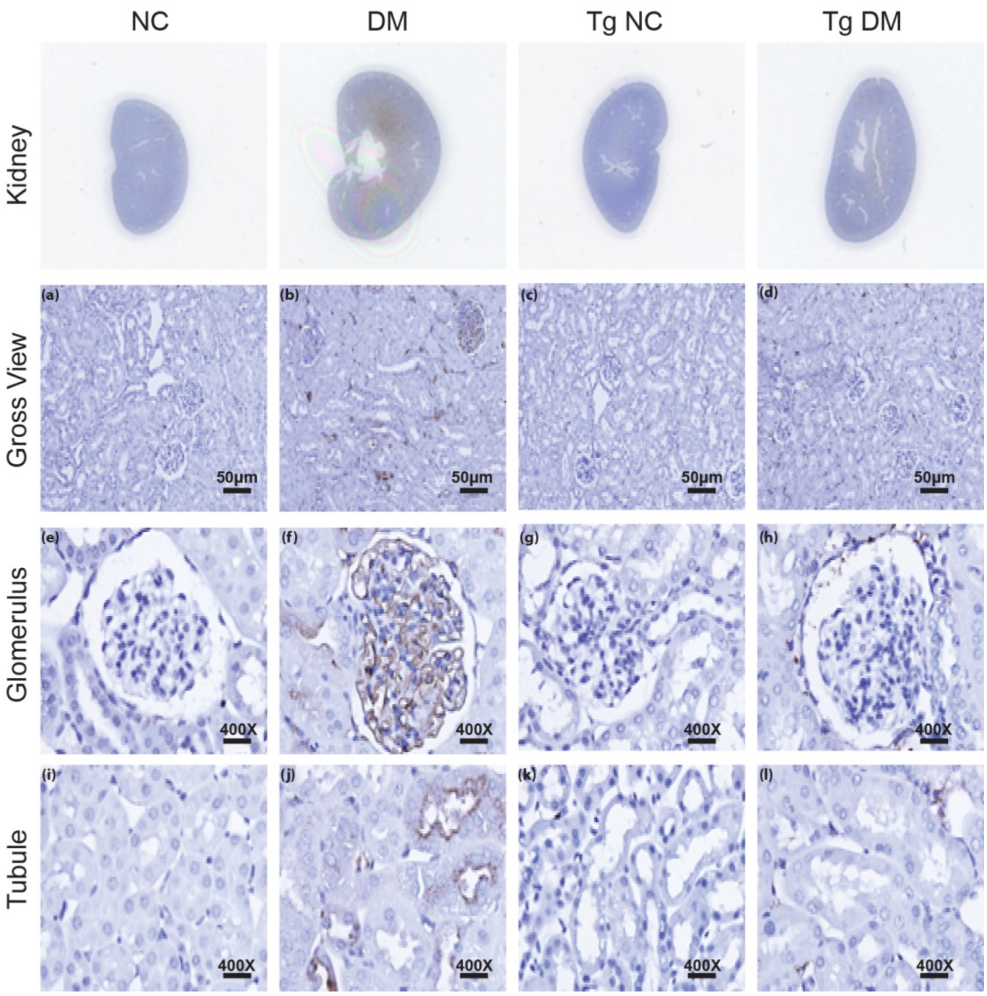

Figure S6. Representative photographs of immunohistochemical staining of endothelial marker CD31 in glomeruli and tubulointerestium of wild type mice and miR-29 transgenic mice without or with STZ treatment at 8 weeks. Representative photomicrographs for Masson's trichrome staining of kidney sections in renal glomeruli and tubulointerestium of the wild-type (WT) and miR-29a transgenic mice (Tg) after induction of diabetes. NC, normal control; DM, diabetes.

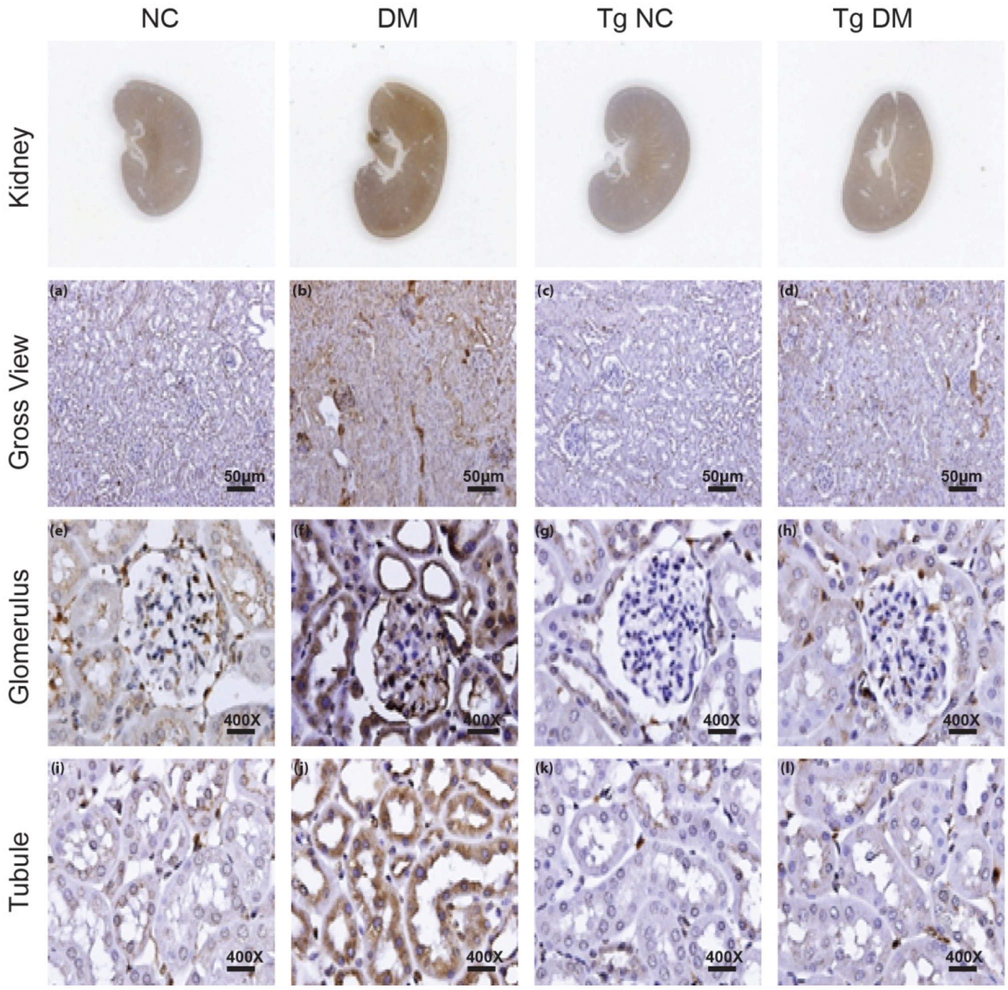

Figure S7. Representative photographs of immunohistochemical staining of endothelial marker (VEGF) in glomeruli and tubulointerstitial of wild type mice and miR-29 transgenic mice without or with STZ treatment at 8 weeks. Representative photomicrographs for VEGF staining of kidney sections in renal glomeruli and tubulointerestium of the wild-type (WT) and miR-29a transgenic mice (Tg) after induction of diabetes. NC, normal control; DM, diabetes.

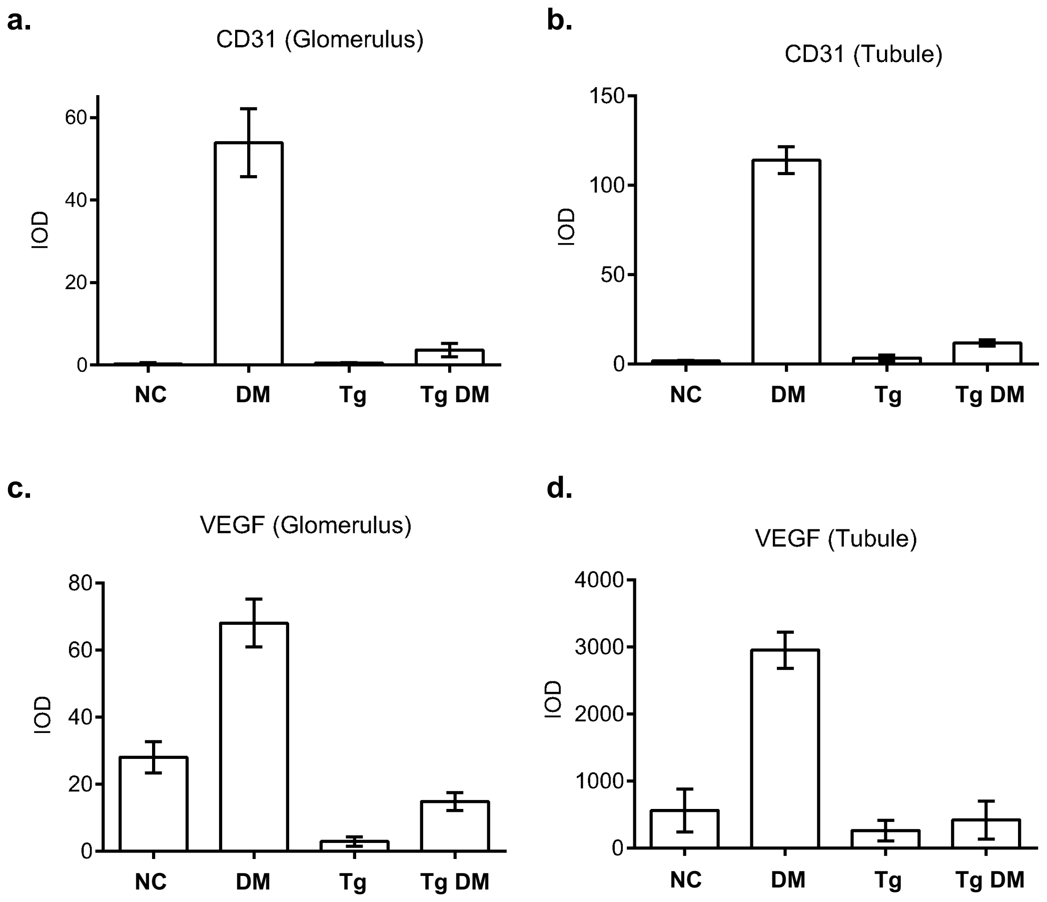

Figure S8. Quantification of fibrotic marker with CD31 and VEGF in wild type mice and miR-29 transgenic mice without or with STZ treatment at 8 weeks. Quantification for CD31 staining of kidney sections in renal glomeruli (a) and tubulointerestium (b); quantification for VEGF staining of kidney sections in the renal glomeruli (c) and tubulointerestium (d) of the wild-type (WT) and miR-29a transgenic mice (Tg) after induction of diabetes. NC, normal control; DM, diabetes.
